# Understanding CD8^+^ T-cell responses toward the native and alternate HLA-A*02:01-restricted WT1 epitope

**DOI:** 10.1038/cti.2017.4

**Published:** 2017-03-17

**Authors:** Thi HO Nguyen, Amabel CL Tan, Sue D Xiang, Anne Goubier, Kim L Harland, E Bridie Clemens, Magdalena Plebanski, Katherine Kedzierska

**Affiliations:** 1 Department of Microbiology and Immunology, University of Melbourne, Peter Doherty Institute for Infection and Immunity, Melbourne, Victoria, Australia; 2 PX Biosolutions, South Melbourne, Victoria, Australia; 3 Department of Immunology and Pathology, Monash University, Melbourne, Victoria, Australia

## Abstract

The Wilms' tumor 1 (WT1) antigen is expressed in solid and hematological malignancies, but not healthy tissues, making it a promising target for cancer immunotherapies. Immunodominant WT1 epitopes, the native HLA-A2/WT1_126-134_ (**R**MFPNAPYL) (HLA-A2/RMFPNAPYL epitope (WT1A)) and its modified variant **Y**MFPNAPYL (HLA-A2/YMFPNAPYL epitope (WT1B)), can induce WT1-specific CD8^+^ T cells, although WT1B is more stably bound to HLA-A*02:01. Here, to further determine the benefits of those two targets, we assessed the naive precursor frequencies; immunogenicity and cross-reactivity of CD8^+^ T cells directed toward these two WT1 epitopes. *Ex vivo* naive WT1A- and WT1B-specific CD8^+^ T cells were detected in healthy HLA-A*02:01^+^ individuals with comparable precursor frequencies (1 in 10^5^–10^6^) to other naive CD8^+^ T-cell pools (for example, A2/HIV-Gag_77-85_), but as expected, ~100 × lower than those found in memory populations (influenza, A2/M1_58-66_; EBV, A2/BMLF1_280-288_). Importantly, only WT1A-specific naive precursors were detected in HLA-A2.1 mice. To further assess the immunogenicity and recruitment of CD8^+^ T cells responding to WT1A and WT1B, we immunized HLA-A2.1 mice with either peptide. WT1A immunization elicited numerically higher CD8^+^ T-cell responses to the native tumor epitope following re-stimulation, although both regimens produced functionally similar responses toward WT1A via cytokine analysis and CD107a expression. Interestingly, however, WT1B immunization generated cross-reactive CD8^+^ T-cell responses to WT1A and could be further expanded by WT1A peptide revealing two distinct populations of single- and cross-reactive WT1A^+^CD8^+^ T cells with unique T-cell receptor-αβ gene signatures. Therefore, although both epitopes are immunogenic, the clinical benefits of WT1B vaccination remains debatable and perhaps both peptides may have separate clinical benefits as treatment targets.

The Wilms' tumor 1 (WT1) gene encodes a zinc-finger transcription factor that has an important role in the differentiation, proliferation and migration of malignant cells.^1, 2, 3^ The gene product, WT1 protein, is expressed in various hematological and solid malignancies^4^ but is negligibly expressed in normal tissues, thus making WT1 an ideal target for cancer immunotherapy strategies.^5^ CD8^+^ T cells are sentinels of the immune system characterized by their ability to detect and kill tumor cells within the tissue and peripheral blood. The *in vivo* efficacy of peptide-induced WT1-specific CD8^+^ T cells to reduce tumor burden has been demonstrated in synergic FBL3 and mWT1-C1498 mice tumor models^6, 7^ and in nude mice inoculated with human tumor cells.^8^ In the latter study, nude mice engrafted with HLA-24^+^ lung cancer cells had a prolonged survival and were able to inhibit cancer cell growth following adoptive transfer of HLA-A24/WT1-specific CD8^+^ T-cell clones. In humans, peptide vaccination studies with HLA-A24/WT1_235-243_ epitopes have been well characterized in the literature to elicit WT1-specific CD8^+^ T-cell responses in adult and children cancer patients.^9, 10, 11, 12, 13^

The HLA-A*02:01 allele is arguably the most common and widespread major histocompatibility complex (MHC) class I allele with up to 60% population coverage in certain regions.^14^ CD8^+^ T-cell responses toward the HLA-A2/WT1_126-134_ RMFPNAPYL epitope (herein called WT1A) have been identified in various HLA-A2^+^ cancer patients. Consequently, recent clinical trials have aimed at boosting the WT1A-specific CD8^+^ T-cell response in cancer patients using WT1A peptide vaccination strategies. Studies have detected an increase in tetramer-positive WT1A-specific CD8^+^ T cells following immunization of leukemia patients using different vaccination platforms including dendritic cell immunotherapy^15, 16, 17^ and peptide-based immunization regimens.^18, 19, 20^ However, the latter studies in acute myeloid leukaemia (AML) or myelodysplastic syndrome (MDS) patients showed that the WT1A-specific CD8^+^ T-cell responses were either short-lived with repeated vaccinations enriching for lower avidity populations,^19^ or could not be further expanded *in vitr*o and may have been functionally impaired following WT1A vaccination.^20^

Pinilla-Ibarz *et al.*^21^ have identified a heteroclitic version of WT1A with an arginine to tyrosine substitution at position 1 (YMFPNAPYL, herein called WT1B). This peptide was shown to bind more stably to HLA-A2 than WT1A^21^ and generated more robust interferon (IFN)-γ-secreting CD8^+^ T-cell responses via ELISPOT. More importantly, WT1B-generated T-cell lines were able to respond to the native WT1A epitope in IFN-γ secretion and cytotoxicity assays using WT1A-pulsed leukemic cell lines as targets.^21^ A number of studies have examined the immunogenicity and biological relevance of the alternate WT1B epitope as a vaccine candidate in AML patients with confirmed expression of WT1 transcripts in bone marrow cells^22^ and patients with lung malignancies.^23^ Maslak *et al.*^22^ reported that vaccination with WT1B peptide induced a significant >2-fold increase in IFN-γ-secreting (via ELISPOT) and WT1A-tetramer^+^CD8^+^ T-cell responses after *in vitro* culture in all three evaluated HLA-A2^+^ patients (out of a possible nine), which could be detected as early as after the third WT1B vaccination. Moreover, CD8^+^ T cells generated by *in vitro* culture with WT1B peptide were cytotoxic against WT1-expressing 697 cancer cells bearing the native epitope, as demonstrated in one patient following vaccination.^22^ In lung cancer patients vaccinated with WT1B (six vaccinations, 12-week period), WT1A-specific CD8^+^ T-cell responses were detected in 5/6 HLA-A2^+^ patients with similar observations.^23^

Despite the safety and clinical feasibility of vaccinating cancer patients with either WT1A or WT1B peptide, it is still unclear whether the alternate WT1B epitope is indeed a more favorable vaccine candidate in terms of its ability to induce or expand an effective polyfunctional WT1-specific CD8^+^ T-cell response in cancer patients. It has also been difficult to directly compare vaccination strategies between WT1A and WT1B, both within individuals (unless in an identical twin setting), and among different HLA-A2^+^ individuals, perhaps due to the extensive diversity in T-cell receptor (TCR) gene usage and the effects of other competing HLA molecules.

Here, for the first time, we detected comparable *ex vivo* naive precursor frequencies of both WT1A- and WT1B-specific CD8^+^ T cells in healthy individuals, and naive WT1A-specific CD8^+^ T cells in HLA-A2.1 mice. We directly compared the immunogenicity of WT1A versus WT1B using a peptide vaccination regimen and found that, albeit both WT1A or WT1B-vaccinated mice could comparably produce robust CD8^+^ T-cell responses to the native WT1A peptide, WT1B-vaccinated mice could also generate cross-reactive CD8^+^ T-cell responses. Importantly, we found that *in vitro* stimulation with WT1A peptide in WT1B-vaccinated mice generated two different cell populations of single-specific WT1A-tetramer^+^CD8^+^ T cells and dually specific WT1A/WT1B-tetramer^+^CD8^+^ T cells bearing uniquely distinct TCR signatures. This finding warrants further investigation into their functional consequences at the molecular level, and in the clinical setting.

## RESULTS

### *Ex vivo* naive WT1-specific CD8^+^ T cells detected in humans and mice

**Figure 1:**
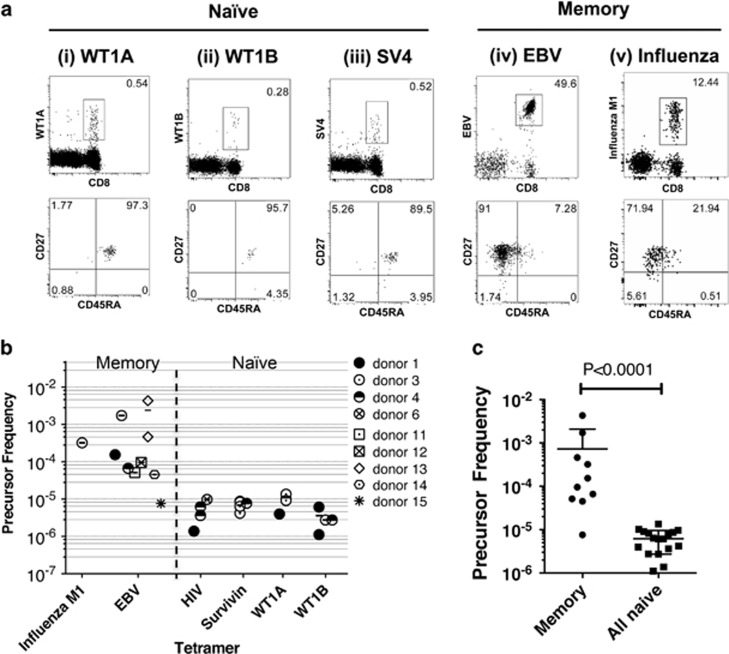
Precursor frequencies of antigen-specific CD8^+^ T cells among healthy HLA-A*02:01^+^ donors. Peripheral blood mononuclear cells (PBMCs) from healthy HLA-A2^+^ donors were enriched for antigen-specific CD8^+^ T cells using TAME. Enrichments were carried out using peptide/MHC tetramers to calculate memory (influenza M1 and EBV) and naive (HIV, survivin, WT1A and WT1B) precursor frequencies. Survivin tetramers were pooled together and included SV3 (*n*=1), SV4 (*n*=2) and SV10 (*n*=1). (**a**) Representative dot plots of one donor of enriched (i–iii) naive and (iv and v) memory populations (gated on CD8^+^ T cells). Lower panel dot plots represent CD27/CD45RA expression profiles from the tetramer population gated directly above. (**b**) Graphed data across *n*=9 donors are shown. (**c**) Graph of pooled data from memory versus naive precursor frequencies. Mean and s.d. are shown.

As naive precursor frequencies can affect the magnitude of effector CD8^+^ T-cell responses and immunodominance hierarchies,^24, 25, 26, 27^ we first assessed whether there were any differences in the naive precursor frequencies between WT1A^+^ and WT1B^+^ CD8^+^ T cells. In healthy individuals, WT1A/B-specific CD8^+^ T cells circulating in the periphery would be classified as ‘naive'. Unlike memory T cells, naive antigen-specific CD8^+^ T cells are present at very low frequencies in the peripheral blood and thus generally fall below the limit of detection with direct *ex vivo* tetramer staining methods. Thus, to enumerate WT1A- or WT1B-specific naive precursors in healthy HLA-A2^+^ individuals, we used a tetramer-associated magnetic enrichment (TAME) method, which increases detection of tetramer-positive CD8^+^ T cells by up to 100-fold.^28, 29, 30^ Naive TAME was performed on HLA-A2^+^ healthy donors (starting with ~30 million peripheral blood mononuclear cells) using peptide/MHC-I tetramers specific for WT1A and the WT1B variant, as well as additional epitopes from other naive antigens (HIV and survivin). Tetramers specific for EBV and influenza A epitopes were used as TAME-positive controls for detection of memory CD8^+^ T-cell populations, which are present in the blood at higher precursor frequencies. For naive enrichments, distinct tetramer^+^CD8^+^ T-cell populations of total CD3^+^ T cells were detected post-enrichment, which mainly co-expressed CD45RA and CD27, markers that are generally characteristic of naive T cells (Figures 1a(i–iii)). Minimal or nil events were detected in the pre-enriched and flow through fractions (Supplementary Figure 1). Cell events higher than 10 were considered above the limit of detection^31, 32^ with a tight clustering of cells and minimal background staining in the CD8^−^ T-cell population for naive post-enrichment fractions. As expected, memory tetramer^+^CD8^+^ T cells were readily detected pre-enrichment, were further enriched following TAME and mainly consisted of a CD27^+^CD45RA^−^ memory phenotype (Figures 1a(iv) and (v)). As expected, there was no overlap between WT1-tetramer-PE^+^ and SV-tetramer APC^+^ populations using combinatorial TAME (Supplementary Figure 1e).

Tetramer-enriched antigen-specific CD8^+^ T-cell precursor frequencies were then calculated based on the total CD8^+^ T-cell population according to Alanio *et al.*^28^ The enriched naive precursor frequencies for HIV, survivin (SV3/4/10), WT1A and WT1B-tetramers were detected at frequencies ranging from 1 in 10^5^–10^6^ of CD8^+^ T cells (Figure 1b), similar to those reported against MART1_26-35_, NY-ESO1_157-165_, HIV-Gag p17_77-85_, HCV NS3_1406-1415_ and HCV Core_132-140_ epitopes.^28^ Conversely, the mean precursor frequency for EBV-specific memory CD8^+^ T cells was expectantly higher at an average of ~1 in 10^3^ CD8^+^ T cells. Thus, using the TAME method, we were able to detect very rare naive WT1A- and WT1B-specific T cells in humans. Interestingly, both WT1A- and WT1B-specific naive CD8^+^ T cells were observed within the same donor with comparable precursor frequencies (donors #1 and #3, Figure 1b), revealing no marked difference between WT1A and WT1B in the naive T-cell pool. Given the limitations of our small sample size in the different naive populations, we performed statistics on pooled memory versus naive precursor frequency data points, which showed that the naive frequencies were significantly lower (*P*<0.0001, non-parametric Mann–Whitney test) in precursor size compared with memory (Figure 1c).

To determine whether there was any overlap between the WT1A and WT1B-tetramer-positive CD8^+^ T-cell populations, we performed naive enrichments using WT1A alone, WT1B alone and both tetramers for donor #3. Single-tetramer enrichments did enrich for both WT1A^+^CD8^+^ T cells (83 cells) and, to a lesser extent, WT1B^+^CD8^+^ T cells (23 cells). However, following dual enrichment, we could only detect WT1A^+^CD8^+^ T cells (113 cells) and minimal numbers of WT1B^+^CD8^+^ T cells (8 cells), which fell below the limit of detection (Supplementary Figure 2). It was possible that these cell populations could have overlapped and competed for tetramer binding because of differential pMHC-class I avidities.

Therefore, further investigation was focused on the mouse model, whereby the WT1 protein is relatively conserved between humans and mice with 96% amino-acid sequence homology. Importantly, the WT1A epitope itself is 100% conserved across mice and humans.^33^ Thus, to compare our findings in humans, we used a similar TAME methodology^27, 31, 32^ to dissect WT1A- and WT1B-specific CD8^+^ T-cell naive precursors in HLA-A2.1 transgenic mice.^34, 35^ CD62L^hi^ WT1A-specific CD8^+^ T-cell naive precursors were observed in 4/5 HLA-A2.1 mice with an average (±s.d.) precursor frequency of 22±4 cells per mouse (*n*=4) and minimal background events of CD62L^lo^WT1A^+^ nonspecific cells (3±1 cells per mouse) (Supplementary Figure 3a). However, CD62L^hi^ WT1B-specific CD8^+^ T-cell naive precursors fell beyond the limit of detection (5±4 cells per mouse, *n*=5), with a high background of CD62L^lo^WT1B^+^ nonspecific cells (18±9 cells per mouse) (Supplementary Figure 3b). Dual naive enrichment with WT1A and WT1B-tetramers showed less detection of CD62L^hi^ WT1A-specific CD8^+^ T cells (2/5 mice) (Supplementary Figure 3ci) and similarly, CD62L^hi^ WT1B-specific CD8^+^ T-cell frequencies were below detection levels with high background staining (Supplementary Figure 3cii).

Given the very low precursor frequencies of these naive cancer-specific CD8^+^ T cells and difficulties in expanding very few naive antigen-specific CD8^+^ T cells *in vitro*, further investigations into the immunogenicity of WT1A versus WT1B and which cell populations were being recruited into the response (that is, cross-reactive versus non-cross-reactive) were conducted by immunizing HLA-A2.1 transgenic mice with both peptides.

### Immunogenicity of WT1A- and WT1B peptide immunization in HLA-A2.1 transgenic mice

**Figure 2:**
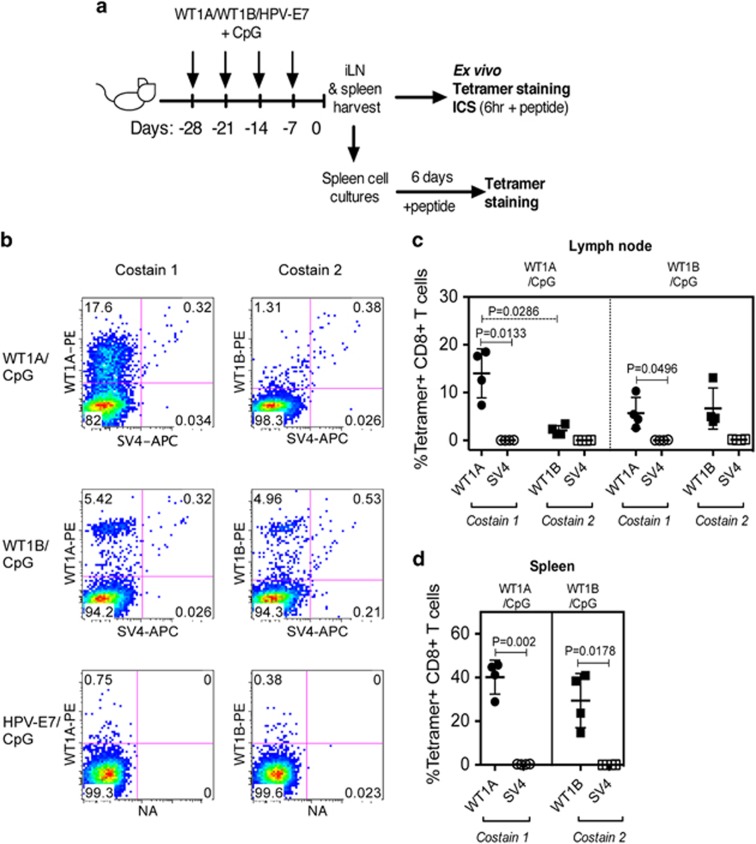
Detection of *ex vivo* WT1A-specific CD8^+^ T-cell responses following WT1A or WT1B vaccination of HLA-A2.1 transgenic mice. Schematic of the vaccination schedule is shown in **a**. In one experiment, mice received four-weekly intradermal injections at the base of tail with WT1A/CpG, WT1B/CpG or the negative control, HPV7-E7/CpG. Seven days following the fourth injection, tetramer staining was performed on iLN or spleen cells. Representative tetramer staining (**b**) in iLN following WT1A/CpG (*n*=4), WT1B/CpG (*n*=4) and control HPV-E7/CpG vaccination (*n*=3). Percentages of tetramer^+^CD8^+^ T cells in iLN (**c**) and spleen (**d**) cells from each vaccinated group are gated on total CD8^+^ T cells (mean±s.d. bars are shown). Reciprocal tetramer staining was not carried out in the spleen (**d**).

HLA-A2.1 transgenic mice have previously been utilized for studying CD8^+^ T-cell responses against WT1A following DNA vaccination.^36^ However, to date, the immunogenicity of WT1B and the direct comparison between WT1A and WT1B vaccination has not yet been investigated. Thus, to compare the immunogenicity between the native WT1A peptide versus the altered WT1B peptide, three groups of HLA-A2.1 transgenic mice were immunized four times weekly with the appropriate peptide in CpG. The vaccination groups included WT1A/CpG and WT1B/CpG, as well as an HPV-E7/CpG group to control for repeated doses of high CpG amounts (Figure 2a). Cells from inguinal lymph nodes (iLNs) (Figure 2b) and spleen from each mouse were isolated and stained with either WT1A-tetramer (co-stain 1) or WT1B-tetramer (co-stain 2) and an irrelevant SV4-tetramer as a negative control.

All WT1A/CpG-vaccinated mice (*n*=4) generated robust WT1A-tetramer^+^CD8^+^ T-cell responses in the iLN (mean±s.d.: 14.02±5.13% of total CD8^+^ T cells, Figure 2c) and spleen (40.15±7.75%, Figure 2d), with negligible staining to the control SV4-tetramer (0.39±0.05%). However, these WT1A/CpG-vaccinated mice generated only minimal WT1B-specific CD8^+^ T-cell responses in the iLN (2.10±1.01%). In contrast, all WT1B/CpG-vaccinated mice (*n*=4) induced both WT1A- and WT1B-tetramer-specific CD8^+^ T-cell responses, with frequencies of 5.72±3.27% and 6.67±4.31% in the iLN, respectively (Figures 2b and c). No significant differences were detected between iLN numbers or tetramer-positive CD8^+^ T cells in the iLN (data not shown). Although WT1B/CpG-vaccinated mice could induce WT1A-tetramer^+^CD8^+^ T cells, the levels were slightly lower compared with WT1A/CpG-vaccinated mice, although this was not significant (data not shown). As expected, WT1A-tetramer or WT1B-tetramer staining was not detected in HPV-E7/CpG-vaccinated mice (Figure 2b).

### WT1B peptide vaccination induces cross-reactive WT1A-specific CD8^+^ T-cell responses

**Figure 3:**
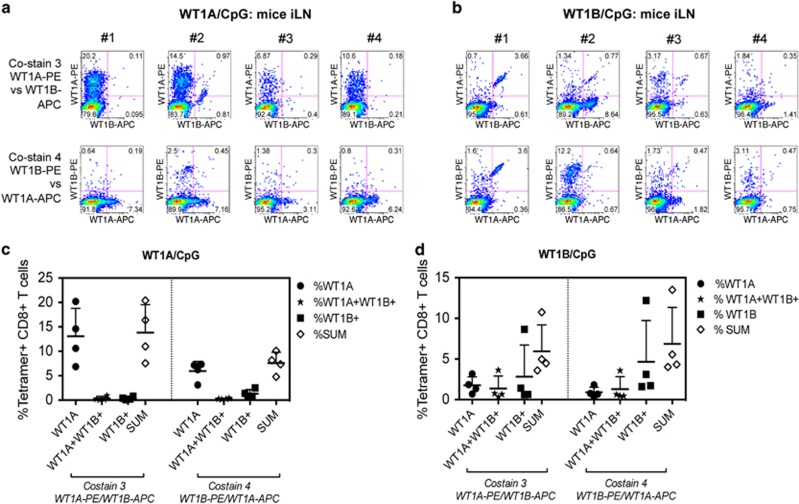
WT1B vaccination induces cross-reactive WT1A-specific CD8^+^ T cells. Seven days following the fourth injection, dual WT1A/WT1B-tetramer staining *ex vivo* was performed in one experiment on iLN cells from four WT1A-vaccinated (**a**) and four WT1B-vaccinated mice (**b**). Individual staining profiles for each mouse are shown using co-stain 3 and co-stain 4. Percentages of tetramer^+^CD8^+^ T cells from WT1A-vaccinated (**c**) and WT1B-vaccinated (**d**) mice are gated on total CD8^+^ T cells (mean±s.d. bars are shown).

We next addressed whether there was any cross-reactivity between the WT1A- or WT1B-specific CD8^+^ T-cell responses induced by either vaccination. We assessed this by co-staining with both WT1A- and WT1B-tetramers using WT1A-tetramer R-phycoerythrin (PE)/WT1B-tetramer allophycocyanin (APC) (co-stain 3) or WT1A-tetramer APC/WT1B-tetramer PE (co-stain 4). In WT1A/CpG-vaccinated mice (Figure 3a), there was very little cross-reactivity detected toward the WT1B-tetramer, and was only observed in one animal (mouse 2) using the more robust tetramer combination for visualizing cross-reactive dual-tetramer^+^ CD8^+^ T cells (co-stain 3).

If selected as a vaccine, WT1B vaccination would ideally be able to induce antitumor CD8^+^ T cells that could recognize the native WT1A epitope presented on cancer cells. Interestingly, in the WT1B/CpG group (Figure 3b), cross-reactivity toward WT1A-tetramer was detected in 4/4 mice, although the extent of overlap varied between animals, including one animal (mouse 4), showing little reactivity to either the individual tetramers or both.

In terms of single and dual-tetramer stains, there was some degree of bias favouring stronger binding to the PE-conjugated tetramer compared with a weaker staining using APC-conjugated tetramer, observed more in the WT1A/CpG-vaccinated group compared with the WT1B/CpG group. However, the cross-reactivity patterns did not change using either co-stains for both vaccinated groups (Figures 3c and d). As such, co-stain 3 was used for further analysis as the WT1A-specific CD8^+^ T-cell response elicited by either vaccine formulations was the main focus of the study.

### Vaccination with WT1A and WT1B peptide induces functionally robust WT1A-specific CD8^+^ T-cell responses

**Figure 4:**
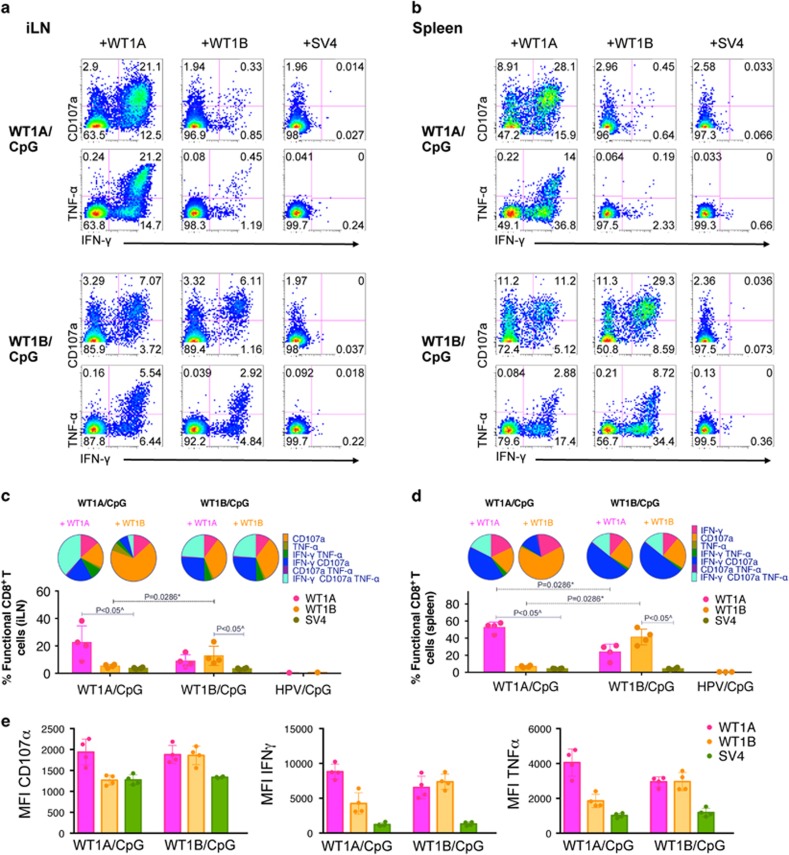
Polyfunctional WT1A-specific CD8^+^ T cells induced following WT1A- or WT1B vaccination. In one experiment, cells isolated from iLN and spleen were stimulated with WT1A, WT1B or the SV4 control peptide in a 6-h intracellular cytokine staining (ICS)-CD107a assay to measure IFN-γ, TNF-α and CD107a responses following vaccination. Representative functional profiles from individual mice in the WT1A/CpG (*n*=4) or WT1B/CpG (*n*=4) are shown for iLN (**a**) and spleen (**b**) as a percentage of CD8^+^ T cells. Polyfunctionality was assessed using Boolean gate analysis to determine the proportion of functional CD8^+^ T cells expressing 1, 2 or 3 of the functional markers in iLN (**c**) and spleen (**d**). Bar graphs below the pie charts show the percentages (mean±s.d.) of total functional cells based on the CD8^+^ T-cell population. Each bar graph dot point represents individual mice, except for dot points in the iLN HPV/CpG group (**c**), which represent WT1A and WT1B peptide responses from pooled mice (*n*=3). (**e**) Mean fluorescence intensity (MFI, geometric mean) of functionally positive CD8^+^ T cells (that is, CD107a^+^, IFN-γ^+^ or TNF-α^+^) of iLN are shown.

To assess the functional quality of WT1A-specific CD8^+^ T-cell responses elicited following either WT1A or WT1B vaccination, cells from iLN and spleens were incubated with either peptide for 6 h and examined for their capacity to induce cytotoxicity, via cell surface expression of the degranulation marker CD107a, and for their cytokine production of IFN-γ and tumor necrosis factor-α (TNF-α). All WT1A- and WT1B-vaccinated mice showed a robust functional CD8^+^ T-cell response toward their cognate peptide stimulus (Figures 4a and b). In the iLN of WT1A/CpG-vaccinated mice stimulated with the cognate WT1A peptide, 22.50±12.10% of the total CD8^+^ T-cell population expressed 1, 2 or 3 of the functional markers (CD107a, IFN-γ or TNF-α), with 38.00±10.95% of total functional cells expressing all three functional markers (Figure 4c). Interestingly, WT1A peptide stimulation in WT1B/CpG-vaccinated mice showed comparable polyfunctionality to its cognate WT1B peptide stimulation, with 23.52±7.34% and 23.76±6.39% of total functional iLN CD8^+^ T cells being IFN-γ^+^CD107a^+^TNF-α^+^, respectively. Notably, similar trends were observed in the spleen (Figure 4d), whereby total functional WT1A-specific CD8^+^ T-cell responses were at least twice times higher following WT1A peptide stimulation in WT1A/CpG-vaccinated mice compared with WT1B/CpG-vaccinated mice, which was statistically significant (*P*=0.0286).

In addition, mean fluorescence intensity (geometric mean) of functionally positive CD8^+^ T cells (that is, CD107a^+^, IFN-γ^+^ or TNF-α^+^) from the iLN was used to measure the output of cytolytic potential (via the CD107a degranulation marker) and cytokines (IFN-γ and TNF-α) produced per cell. Following WT1A peptide stimulation (Figure 4e), the CD107a mean fluorescence intensities were similar between both WT1A- and WT1B-vaccinated mice groups, whereas the cytokine mean fluorescence intensities were slightly lower for the WT1B-vaccinated mice, however, this was not significant, suggesting that WT1A peptide stimulation yielded functionally comparable responses between the two vaccinated mice groups. In all WT1-vaccinated mice, functional CD8^+^ T-cell responses to the irrelevant SV4 peptide were minimal (<1%) and WT1A- and/or WT1B-specific CD8^+^ T-cell responses were not detected in the HPV-E7/CpG-vaccinated group.

### WT1A-specific CD8^+^ T cells expand in culture from WT1A- or WT1B-vaccinated mice

**Figure 5:**
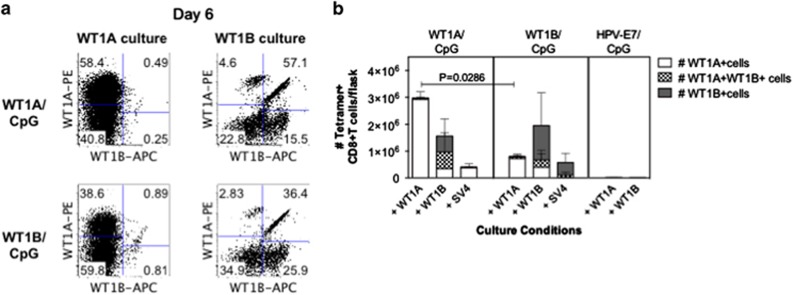
Expansion of cross-reactive WT1A-specific CD8^+^ T cells. In one experiment, splenocytes from mice immunized with WT1A (*n*=4), WT1B (*n*=4) or an irrelevant HPV peptide (*n*=3) were cultured with WT1A, WT1B or SV4 peptides in the presence of IL-2. After 6 days of culture, cells were costained with WT1A-tetramer-PE and WT1B-tetramer APC (co-stain 3). Representative tetramer co-staining profiles (**a**) of individual mice cultured with WT1A and WT1B peptides from both WT1A/WT1B-vaccinated groups. Bar graph (**b**) showing absolute numbers of expanded single-tetramer^+^ and dual-tetramer^+^ CD8^+^ T cells (mean±s.d. are shown).

WT1A peptide vaccination trials in AML and MDS patients have found that WT1A-specific CD8^+^ T-cell responses are short-lived and expand poorly following *in vitro* culture or repeated vaccination.^19, 20^ In addition, it is very difficult to directly compare the *de novo* efficacy of different vaccine formulations within the same individual. To compare the proliferative capacity of CD8^+^ T cells following WT1A versus WT1B vaccination in our mouse system, 2–3 × 10^7^ splenocytes per mouse from each group were expanded for 6 days in the presence of WT1A, WT1B or the control SV4 peptide, before determining tetramer specificity based on co-stain 3 (WT1A-tetramer PE versus WT1B-tetramer APC) (Figure 5a). The cultures generated with the SV4 peptide served as control cultures from the vaccination groups. In addition, cells from the HPV-E7/CpG vaccination group were stimulated with the WT1A and WT1B peptide and as expected, no expansion was detected confirming the absence of nonspecific expansion by these peptides.

In the WT1A/CpG-vaccinated group, cognate WT1A peptide stimulus resulted in the predominant and selective expansion of single WT1A-tetramer^+^CD8^+^ T cells and was on average 9.4-fold higher (range=5.0–18.6-fold) in numbers compared with the SV4 control culture, which consisted mainly of single WT1A-tetramer^+^CD8^+^ T cells (Figure 5b). Substantially lower numbers of double WT1A/WT1B-tetramer^+^ or WT1B-tetramer^+^cells were observed following WT1A peptide stimulation. In contrast, stimulation with WT1B peptide expanded varying levels of double WT1A/WT1B-tetramer^+^ and single WT1B-tetramer^+^CD8^+^ T cells, but there was no further expansion of single WT1A-tetramer^+^ cells when compared with the SV4 control group.

In the WT1B/CpG-vaccinated group, stimulation cultures with the control SV4 peptide mainly consisted of single WT1B-tetramer^+^CD8^+^ T cells and a smaller proportion of double WT1A/WT1B-tetramer^+^CD8^+^ T cells. Importantly, WT1A peptide stimulus predominantly expanded single WT1A-tetramer^+^ CD8^+^ T cells that were otherwise undetectable in the SV4 control culture. Stimulation with the WT1B peptide also enabled the expansion of single WT1A-tetramer^+^CD8^+^ T cells in 2/4 mice, as well as a low two- to six-fold expansion of double WT1A/WT1B-tetramer^+^CD8^+^ T cells.

In terms of the expansion potential of cells generated from vaccination, the number of WT1A-tetramer^+^CD8^+^ T cells was significantly ~4-fold higher in the WT1A-vaccinated group compared with the WT1B-vaccinated group following WT1A peptide stimulation (*P*=0.0286, Figure 5b). Our data suggest that WT1A vaccination can generate cells with a higher *in vitro*-peptide-driven expansion potential.

### Cross-reactive and non-cross-reactive WT1-specific CD8^+^ T cells have distinct TCR signatures

**Figure 6:**
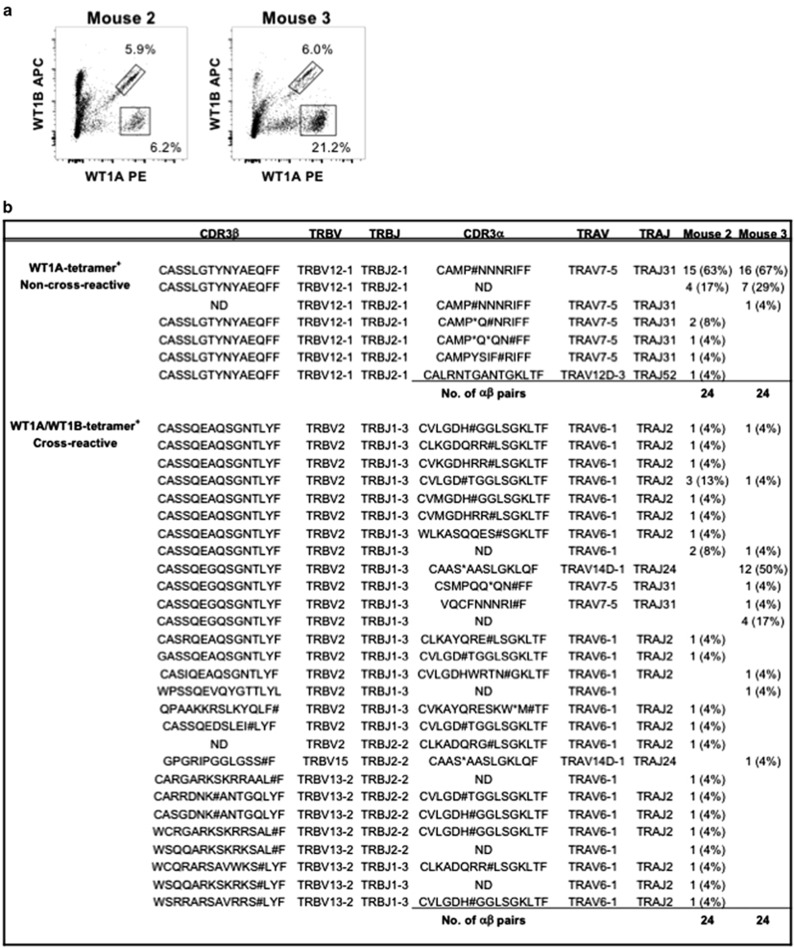
WT1-specific TCRαβ repertoires in WT1B-vaccinated mice following WT1A *in vitro* stimulation. Sort profiles of mouse 2 and 3 (**a**) after splenocytes were *in vitro*-cultured for 7 days with WT1A peptide from WT1B-vaccinated mice. Cells were single-cell sorted based on viable, WT1A^+^ non-cross-reactive or cross-reactive WT1A^+^WT1B^+^ CD8^+^ T cells before performing TCR analysis to determine the CDR3αβ regions and frequency (**b**) for non-cross-reactive and cross-reactive clonotypes from each mouse. ND, not determined; X, undefined amino acid; #, insertion or deletion causing an out-of-frame shift thereby producing an unproductive TCR chain; *, stop codon, which also gives rise to an unproductive TCR chain.

To further explore the different cell populations of single-reactive (WT1A^+^) versus cross-reactive (WT1A^+^WT1B^+^) WT1-specific CD8^+^ T cells induced by WT1B vaccination, single-cell paired TCRαβ analysis was performed on splenocytes cultured for 7 days with WT1A peptide from WT1B-vaccinated mice. Single-cell populations were sorted based on WT1A^+^ and WT1A^+^WT1B^+^ CD8^+^ T cells from mouse 2 and 3 (Figure 6a), showing two different TCRαβ signatures. For WT1A^+^ CD8^+^ T cells, the TCR repertoires were highly clonal and near-identical between mouse 2 and 3, with almost all clonotypes bearing a productive TRBV12-1/TRBJ2-1 TCRβ chain with a CDR3β sequence of CASSLGTYNYAEQFF (Figure 6b). A non-productive TCRα chain was predominantly observed for both mice bearing TRAV7-5/TRAJ31 TCRα genes. The cross-reactive TCR signatures in mouse 2 and 3 were commonly bearing a TRBV2/TRBJ1–3 TCRβ chain with a non-productive TRAV6-1/TRAJ2 TCRα chain (Figure 6b). Although these TCR signatures are quite distinct, we cannot rule out the possibility that these two different cell populations share a similar productive TCRα chain, which we were unable to amplify. Taken together, our data show that WT1B vaccination followed by WT1A re-stimulation induces two different cell populations of single and cross-reactive WT1-specific CD8^+^ T cells bearing uniquely distinct TCR signatures. This allows for further insights into measuring their functional avidity, as well as eventually assessing whether WT1B vaccination offers a better clinical option, by virtue of having the ability to induce cross-reactive WT1-specific CD8^+^ T cells.

## DISCUSSION

WT1 is a promising target antigen for anticancer vaccination and adoptive immunotherapy strategies given its widespread expression in various human malignancies. In this study, we used tetramer-associated enrichment strategies to detect both WT1A- and modified WT1B-specific CD8^+^ T cells of similar *ex vivo* precursor frequencies in healthy individuals, as well as detecting naive precursors of WT1A-specific CD8^+^ T cells in HLA-A2.1 transgenic mice. We directly compared the immunogenicity of the native WT1A epitope and the modified WT1B epitope in a HLA-A2.1 transgenic mice model and showed that both vaccination regimens were capable of inducing robust WT1A-specific CD8^+^ T cells responses, although WT1B vaccination had the further ability of inducing cross-reactive WT1A/WT1B-specific CD8^+^ T cells.

This is, to our knowledge, the first time that WT1A-specific CD8^+^ T cells have been detected in healthy individuals (and WT1B-specific) and in HLA-A2.1 mice *ex vivo*. Our studies indicated that the precursor frequency of WT1A and the heteroclitic WT1B were comparable in humans. Further studies would be required to determine whether the naive precursors represent overlapping or distinct CD8^+^ T-cell precursors. Our investigations in mice could not conclusively address this as we were unable to detect any naive precursors of WT1B-specific CD8^+^ T cells via TAME. A recent human study by Schmied *et al.*^37^ has reported the *ex vivo* detection of naive CD8^+^ T cells specific for another WT1 epitope, HLA-A2/WT1_37-45_ (VLDFAPPGA), using similar tetramer enrichment strategies. They included samples (*n*=10) with at least 5 tetramer^+^CD8^+^ events (our study's cut-off was at least 10 events), and showed comparable precursor frequencies averaging 1 in 10^5^–10^6^ cells within the CD8^+^ T-cell compartment. We acknowledge that it would be very useful to perform comparative TAME analyses between our WT1A and WT1B antigens with other published cancer antigens, such as WT1_37-45_ from Schmied *et al.'s* and MART1_26-35_ from Alanio *et al.'s* studies.^28, 37^ Nevertheless, these data on numbers of naive CD8^+^ T cells directed against WT1A and WT1B can be used for the future assessments of any cancer vaccine, which utilizes WT1A and/or WT1B components. Although our donor numbers are small, the main focus of our study was to ultimately compare the CD8^+^ T-cell recruitment and immunogenicity between WT1A and WT1B, and hence directly assess the immunological relevance of WT1B in future peptide vaccination strategies.

WT1A-specific CD8^+^ T cells can be detected after *in vitro* expansion in healthy HLA-A2^+^ donors,^37^ yet the ability to induce effective WT1A-specific CD8^+^ T-cell responses in cancer patients has been less successful in WT1A-targeted vaccination studies. A study by Van Tendeloo *et al.*^15^ showed that tetramer^+^CD8^+^ T-cell populations specific for WT1A (and other HLA-A2/WT1 epitopes: WT1_37-45_, WT1_187-195_ and WT1_235-243_)^16^ were induced in 2/5 patients (>1.5-fold percentage increase in the blood) following vaccination with dendritic cells loaded with WT1 tumor RNA, which significantly correlated with long-term responses to DC immunotherapy.^15^ In another trial involving six biweekly subcutaneous injections of WT1A peptide, low WT1A-tetramer^+^CD8^+^ T-cell responses were detected *ex vivo* in 7/7 AML or MDS patients after the first vaccine dose (median 0.1% of CD8^+^ T cells, range 0.07–0.19%).^18, 19^ However, these responses were short-lived and not detected in the peripheral blood 3 months following the sixth vaccine dose in all 6/6 patients evaluated, with all the WT1A-specific CD8^+^ T-cell responses becoming increasingly of lower avidity following repeated vaccinations,^19^ thus suggesting that these cells had limited functionality. Similarly in Uttenthal *et al.*'s^20^ study, WT1A-tetramer^+^CD8^+^ T-cell responses were observed in 6/8 AML patients following five cycles of 3-weekly doses of WT1A peptide vaccination. Yet, these responses could not be further expanded *in vitr*o, again indicating an impairment of responses following WT1A vaccination.^20^ Given the weak responses elicited with WT1A vaccination, other groups have focussed on the modified WT1B epitope as an alternative strategy to induce anti-WT1 tumor responses in cancer patients.^22, 23^ However, there is still a lack of evidence to suggest that WT1B is superior to WT1A for use in anticancer vaccination trials.

A proof of principle for WT1 epitope modification to enhance anti-WT1 CD8^+^ T-cell responses has been well established in HLA-A24^+^ cancer patients whereby the modified Y236M-variant of the HLA-A24/WT1_235-243_ (CYTWNQMNL) epitope has been used in a number of peptide vaccination clinical trials involving both adults and children with hematological and solid cancers in Japan,^9, 10, 11, 12, 13^ with some patients within each study having positive clinical outcomes associated with an increase in WT1-specific CD8^+^ T-cell responses. For example, patients with various malignancies (that is, breast, lung and renal cancers, MDS and AML) were able to induce WT1-tetramer^+^CD8^+^ T-cell responses following vaccination with modified WT1_235-243_ peptide vaccine formulated with Montanide adjuvant.^10^ This correlated with a positive clinical response in the form of primary and metastatic tumor regression, reduced WT1/CEA marker expression or reduced leukemic cells, in 12/20 patients.^10^

In this study, our humanized mice model was used to assess whether the WT1B epitope offers any advantages over the native WT1A epitope in a vaccine strategy. HLA-A2/A24-transgenic mice have been used recently for preclinical testing of HLA-A2/WT1_126-134_ and HLA-A24/WT1_235-243_ epitopes for further development of WT1-targeted immune therapies, but no comparisons were made with the variant WT1B epitope and the majority of their work focused more toward the HLA-A24/WT1_235-243_-specific CD8^+^ T-cell response.^38^ Our findings suggest, for the first time, that peptide vaccination with both WT1A and WT1B epitopes generates a comparable proportion of *de novo* tetramer^+^CD8^+^ T-cell responses toward their cognate antigen. The increase in stability of the HLA/peptide complex with the alternate WT1B peptide did not necessarily result in stronger WT1B-specific CD8^+^ T-cell responses, suggesting that the size or the capacity of WT1A- and WT1B-specific T cells to be recruited and mobilized from the naive repertoires are not equal. However, WT1B vaccination did have the ability to induce cross-reactive WT1A^+^WT1B^+^ CD8^+^ T cells, which comprised a unique TCRαβ signature that was distinct from the single-reactive WT1A^+^ TCR signature. More in-depth dissection of CD8^+^ TCR repertoires targeting WT1A or both WT1A/WT1B epitopes will be important for understanding whether the cross-reactive T-cell clones expanded upon the initial WT1B vaccination are of higher functional avidity than those of CD8^+^ T cells elicited by WT1A vaccination. Similar observations have been observed with two altered peptides, derived from wild-type HIV-reverse transcriptase_309-317_ peptide (ILKEPVHGV), whereby I1F and I1Y variants increased the cell surface half-lives of the HLA/peptide complexes by >3-fold over the wild-type complex but had differing immunogenicity profiles.^39^

Of biological relevance, we were able to detect WT1A-tetramer^+^CD8^+^ T-cell responses *ex vivo* from WT1B-vaccinated mice, and even more so following WT1A peptide stimulation *in vitro*, which are important findings given that WT1A is the native tumor target. This suggests that in a healthy or naive scenario, WT1A vaccination could be effective at generating *de novo* responses in a prophylactic setting. In a cancer patient setting, however, WT1A or WT1B vaccination would serve to expand pre-existing populations or recruit cross-reactive CD8^+^ T-cell responses, respectively, and as discussed, previous studies indicated that WT1A vaccination in AML and MSD patients yielded functionally limited responses. Interestingly, WT1B vaccination was able to induce a population of functionally cross-reactive WT1A^+^WT1B^+^ CD8^+^ T cells, which encompassed a unique TCRαβ signature that was different to those of single-reactive WT1A^+^CD8^+^ T cells. In support, binary structures of the native and modified WT1 peptide in complex with the HLA-A2 molecule have indicated that the modified peptide alters the positions of the charged side chains, which may impact on TCR recognition.^40^ Whether the cross-reactive CD8^+^ T-cell population induced by WT1B is of a higher pMHC-class I avidity to the single-reactive T-cell population remains to be addressed. This would help to understand whether WT1B is a better candidate for human clinical trials.

Our findings support the utility of WT1B vaccination as a way to elicit not only CD8^+^ T cells that react to the native tumor epitope, but also to induce a unique population that does not overlap with exclusively WT1A-specific CD8^+^ T cells, given that exclusive WT1A expansion induced poorly functional or short-lived cells in humans.^19, 20^ In addition, previous studies have shown that the WT1B peptide elicits more robust IFN-γ ELISPOT CD8^+^ T-cell responses in humans than the native WT1A peptide.^21, 41^ However, in our humanized mice model, we found no significant differences in the polyfunctional capacity of WT1A-specific CD8^+^ T cells derived from either WT1A- or WT1B-vaccinated mice groups.

Overall, we compared the immunogenicity of WT1A versus the altered WT1B epitope using a humanized mice model and demonstrated that both WT1A and WT1B vaccination generated functionally similar CD8^+^ T-cell responses to the cognate antigen *ex vivo*, and both vaccination regimens could be readily expanded in response to the cognate peptide. Although WT1A generated greater WT1A-specific CD8^+^ T-cell responses, WT1B had the potential to generate a proportion of dual responses that cross-reacted with WT1A, and could be expanded by the WT1A peptide. Based on our findings in mice and the presence of both WT1A- and WT1B-specific naive CD8^+^ T-cell precursors in healthy humans, it is still debatable whether WT1A or WT1B epitopes are suitable candidates for consideration in future prophylactic vaccination and adoptive immunotherapeutic strategies.

## METHODS

### Human subjects

Human experimental work was conducted according to Declaration of Helsinki principles and approved by the Human Ethics Committee (University of Melbourne, Parkville, VIC, Australia; ethics ID #0931311.5). Buffy packs from healthy donors were obtained from the Australian Red Cross Blood Service (ARCBS) (Melbourne, VIC, Australia) with informed consent. Peripheral blood mononuclear cells were isolated by Ficoll-Paque (GE Healthcare, Uppsala, Sweden) density gradient centrifugation and cryopreserved at −196 °C until required in our laboratory or at the Burnet ImmunoMonitoring Facility (Burnet Institute, Melbourne, VIC, Australia). HLA class I molecular typing was performed by the Victorian Transplantation and Immunogenetics Service (ARCBS, West Melbourne, VIC, Australia) using the Luminex platform and microsphere technology (One Lambda, Canoga Park, CA, USA), with LABType SSO HLA typing kits (One Lambda).

### TAME of human epitope-specific CD8^+^ T cells

Approximately 20–50 million cryopreserved peripheral blood mononuclear cells were thawed and incubated with anti-human FcR block (Miltenyi Biotec, Bergisch Gladbach, Germany) for 15 min on ice. Cells were then stained with PE- and/or APC-tetramer for 1 h, washed once, then incubated with anti-PE and/or anti-APC microbeads (Miltenyi Biotec) before passing through a LS column (Miltenyi Biotec) to enrich for tetramer^+^ cells as previously described.^30, 31^ Cells were then surface stained with human anti-CD3 BV570 (#300436, Biolegend, San Diego, CA, USA), anti-CD8 PerCP-Cy5.5 (#565310, BD Biosciences, San Jose, CA, USA), anti-CD14 APC-H7 (#560180, BD Biosciences), CD19 APC-H7 (#560177, BD Biosciences), anti-CD45RA FITC (#555488, BD Biosciences), anti-CD27 AF700 (#56027942, eBioscience, San Diego, CA, USA) and LIVE/DEAD Fixable Aqua (Molecular Probes, Eugene, OR, USA) before fixing with 1% paraformaldehyde. Cells were acquired using the LSR Fortessa II (BD Biosciences) and analyzed by FlowJo software (Treestar, Inc., Ashland, OR, USA).

### HLA-A2.1 transgenic mice

HHD HLA-A2.1 transgenic mice were developed by Dr François Lemonnier^35^ and the parental pairs were provided by the Pasteur Institute, Paris, France. HLA-A2.1 mice express a chimeric monochain (HHD molecule) of the human β2m in its mature from, covalently linked to the α1-α2 domains of HLA-A*02:01 and the α3, cytoplasmic and transmembrane domains of mouse H-2D^b^ on a double-knockout H-2D^b^^−/−^ β2m^−/−^ C57BL/6 mouse background.^34, 35^ Female/male HLA-A2.1 mice aged 6–12 weeks were obtained from the BioResources Facility (Department of Microbiology and Immunology, The University of Melbourne). The TAME mice experiments were approved and conducted under guidelines set by the University of Melbourne Animal Ethics Committee (ethics approval number 1312880.6). For the vaccination experiments, mice were housed in the Alfred Medical Research and Education Precinct (AMREP) Animal Facility (Melbourne, VIC, Australia) and experimental protocols were approved by the AMREP Animal Ethics Committee (ethics approval number E1051/2011/M).

### Peptides, immunogens and tetramers

BMLF1_280-288_-GLCTLVAML (EBV), GAG_77-85_-SLYNTVATL (HIV), Survivin_95-104_ (ELTLGEFLKL) (SV3), Survivin_96-104_-LTLGEFLKL (SV4), Survivin_96-104_ variant-LMLGEFLKL (SV10) and WT1_126-134_-RMFPNAPYL (WT1A) peptides were synthesized by Mimitopes (Notting Hill, VIC, Australia). WT1A was also synthesized by CS Bio (Menlo Park, CA, USA). WT1_126-134_ variant-YMFPNAPYL (WT1B) and HPV-E7_86-93_-TLGIVCPI (HPV-E7) was from Auspep (Tullamarine, VIC, Australia) and influenza A M1_58-66_-GILGFVFTL was from Genscript (Piscataway, NJ, USA). CpG oligonucleotide (ODN 2395) (Invivogen, San Diego, CA, USA), herein referred to as CpG, was used as adjuvant in the peptide vaccine formulations. Peptides were refolded with HLA-A*02:01 α-heavy chain-BirA and β2-microglobulin to generate monomers (ImmunoID, University of Melbourne), before conjugating with streptavidin–R-phycoerythrin or streptavidin–allophycocyanin (BD Biosciences) to form tetramers.^42^

### Test material and immunizations

Peptide vaccine formulations were made by individually mixing WT1A, WT1B or the control peptide HPV-E7 (1 mg ml^–1^) with CpG (0.4 mg ml^–1^) in phosphate-buffered saline buffer. HLA-A2.1 mice were randomly divided into groups of four and immunized with 50 μl of peptide/CpG formulation intradermally at the base of tail four times, weekly apart, in a randomized non-blinded study. Draining iLNs and spleens were harvested 7 days following the fourth weekly injection.

### Tetramer staining of mice cells

Freshly isolated mouse lymph nodes and splenocytes or cultured splenocytes were stained with tetramer for 60 min at room temperature, washed twice, then labeled with mouse anti-CD8 PacBlue (#558106, BD Biosciences), anti-CD3 PerCP-Cy5.5 (#551163, BD Biosciences), anti-CD44 APC-Cy7 (#103028, Biolegend), anti-CD62L Pe-Cy7 (#104418, Biolegend), anti-KLGR1 FITC (#11589382, eBioscences) and LIVE/DEAD Fixable Aqua. Cells were then fixed and analyzed by flow cytometry.

### Naive TAME in mice

TAME was performed as previously described^31, 32^ in HLA-A2.1 transgenic mice. Briefly, single-cell suspensions of pooled spleen and lymph nodes were stained with WT1A-PE, WT1B-APC or both tetramers before magnetic enrichment. Mice were randomly divided into groups of five. Enriched cells were then stained with mouse anti-CD3 PerCP-Cy5.5, anti-CD8 APC-Cy7 (#557654, BD Biosciences), anti-CD44 PE-Cy7 (#25044182, eBiosciences), anti-CD62L BV570 (#104433, Biolegend), anti-CD4 FITC (#553055, BD Biosciences), anti-B220 FITC (#553088, BD Biosciences), anti-F4/80 FITC (#11480185, eBiosciences), anti-CD11b FITC (#11011282, eBiosciences), anti-CD11c FITC (#11011482, eBiosciences), anti-I-A^b^ FITC (#116406, Biolegend) and LIVE/DEAD Fixable Aqua before acquiring using the LSR Fortessa II.

### *In vitro* re-stimulation cultures

Mouse splenocytes were re-stimulated *in vitro* as described.^43^ Briefly, 2–3 × 10^7^ spleen cells from each mouse were cultured for 6 days in the presence of 10 U ml^−1^ recombinant human IL-2 (PeproTech, Rocky Hill, NJ, USA) at a stimulator to effector ratio of 1:3. Stimulator cells were pulsed with 10 μg ml^−1^ peptide for 90 min at 37 °C, then washed twice before returning to effector cells.

### Intracellular cytokine staining assay

Single-cell suspensions of mouse spleen and lymph node cells were processed as previously described.^44^ Intracellular cytokine staining was performed using 1–3 × 10^6^ cells as described in Tan *et al.*^44^ with the following changes. Cells were cultured for 6 h in the presence of GolgiPlug and GolgiStop (BD Biosciences).

### Single-cell reverse transcriptase-PCR and sequencing

Splenocytes *in vitro* cultured for 7 days with WT1A peptide from WT1B-vaccinated mice were stained with tetramer and cell surface mAbs before single WT1A-specific or cross-reactive WT1A/WT1B-specific CD8^+^ T cells were sorted individually into 96-well Twin.tec PCR plates (Eppendorf, Hamburg, Germany) using a BD FACSAria III (BD Biosciences). Analysis of paired CDR3α and CDR3β regions were performed by multiplex-nested reverse transcriptase PCR before sequencing of TCRα and TCRβ products, essentially as described.^32, 45, 46^ Sequences were analyzed according to the IMGT/V-QUEST web-based tool.^47, 48^

### Statistical analysis

Statistical analysis was carried out using GraphPad Prism software (San Diego, CA, USA). Paired non-parametric analysis of variance with *post-hoc* Dunn's multiple comparison test was performed and differences were considered significant when *P*-values were ⩽0.05 with a 95% confidence level.
