# Supplementary material for: Understanding CD8+ T-cell responses toward the native and alternate HLA-A*02:01-restricted WT1 epitope
Source: Clin Transl Immunology. 2017 Mar 17;6(3):e134–. doi: 10.1038/cti.2017.4 (PMC5382434; doi:10.1038/cti.2017.4)
Supplement: Supplementary Information [file cti20174x1.docx]

**Supplementary Information: Nguyen *et al.***

**Supplementary Methods**

***HLA-A24.2 transgenic mice***

HHD HLA-A24.2 transgenic mice were developed by Dr François Lemonnier^1^ and the parental pairs were provided by the Pasteur Institute. HLA-A24.2 mice expressed a chimeric monochain of the human β2m in its mature from, covalently linked to the α1-α2 domains of HLA-A*24:02 and the mouse H2D^b^ α3, cytoplasmic and transmembrane domains, on a triple-knockout H-2D^b^-/- H-2K^b^-/- β2m-/- C57BL/6 mouse background.^1^ Female/male HLA-A24.2 mice aged 6-12 weeks were obtained from the BioResources Facility (Department of Microbiology and Immunology, The University of Melbourne). All animal experiments were approved and conducted under guidelines set by the University of Melbourne Animal Ethics Committee (ethics approval number 1312880.6).

**Supplementary Figures**

**Supplementary Figure 1.** Detection of naïve antigen-specific CD8^+^ T cells via TAME. PBMCs from healthy HLA-A2^+^ donors were enriched for antigen-specific CD8^+^ T cells using TAME. Upper panel dot plots show representative staining of pre-enriched, post-enriched and the flow through fractions following TAME with the indicated tetramers (**a-c**), with EBV tetramer as a positive control (**d**). Cell populations were gated on CD3^+^ T cells. Lower panel dot plots represent CD27/CD45RA expression profiles from the tetramer population gated directly above (**a-d**). Representative post-enriched dot plot of one donor (gated on CD8^+^ T cells) showing no overlap between the different antigen-specific CD8^+^ T cell populations using PE- (x-axis) and APC- (y-axis) tetramers (**e**).

**Supplementary Figure 2.** Competitive tetramer binding between HLA-A*02:01-restricted WT1A and WT1B epitopes. PBMC from Donor #3 were stained with WT1A-tetramer (**a**), WT1B-tetramer (**b**) or both (**c**) before performing TAME. Cells were gated on CD3^+^ T cells and represented post-enriched fractions. Bottom panels represented the CD27 versus CD45RA expression profiles. Loss of WT1B-specific CD8^+^ T cells was evident when both WT1 tetramers were combined, as shown in **c** and **d** (gated on CD8^+^ T cells).

**Supplementary Figure 3.** Enumeration of naïve precursors of WT1-specific CD8^+^ T cells in mice. Single-cell suspensions of spleen and lymph nodes per mouse (n=5 per group) were stained with WT1A-tetramer (**a**), WT1B-tetramer (**b**) or both (**c**) before performing TAME. Representative dot plots of pre-enriched, post-enriched and flow through fractions are shown. The number of events for post-enriched fraction is indicated within the dot plot. Cells were gated on live, dump channel (FITC)-negative CD8^+^ T cells. As a negative control, each HLA-A24.2 mouse was stained with WT1A-tetramer (**d**) or WT1B-tetramer (**e**) in which dot plots from the post-enriched fraction are shown.

**Supplementary References**

1. Boucherma R, Kridane-Miledi H, Bouziat R, Rasmussen M, Gatard T, Langa-Vives F *et al.* HLA-A*01:03, HLA-A*24:02, HLA-B*08:01, HLA-B*27:05, HLA-B*35:01, HLA-B*44:02, and HLA-C*07:01 monochain transgenic/H-2 class I null mice: novel versatile preclinical models of human T cell responses. *J Immunol* 2013; **191:** 583-593.
