# Supplementary figures and images for: Understanding CD8+ T-cell responses toward the native and alternate HLA-A*02:01-restricted WT1 epitope
Source: Clin Transl Immunology. 2017 Mar 17;6(3):e134–. doi: 10.1038/cti.2017.4 (PMC5382434; doi:10.1038/cti.2017.4)

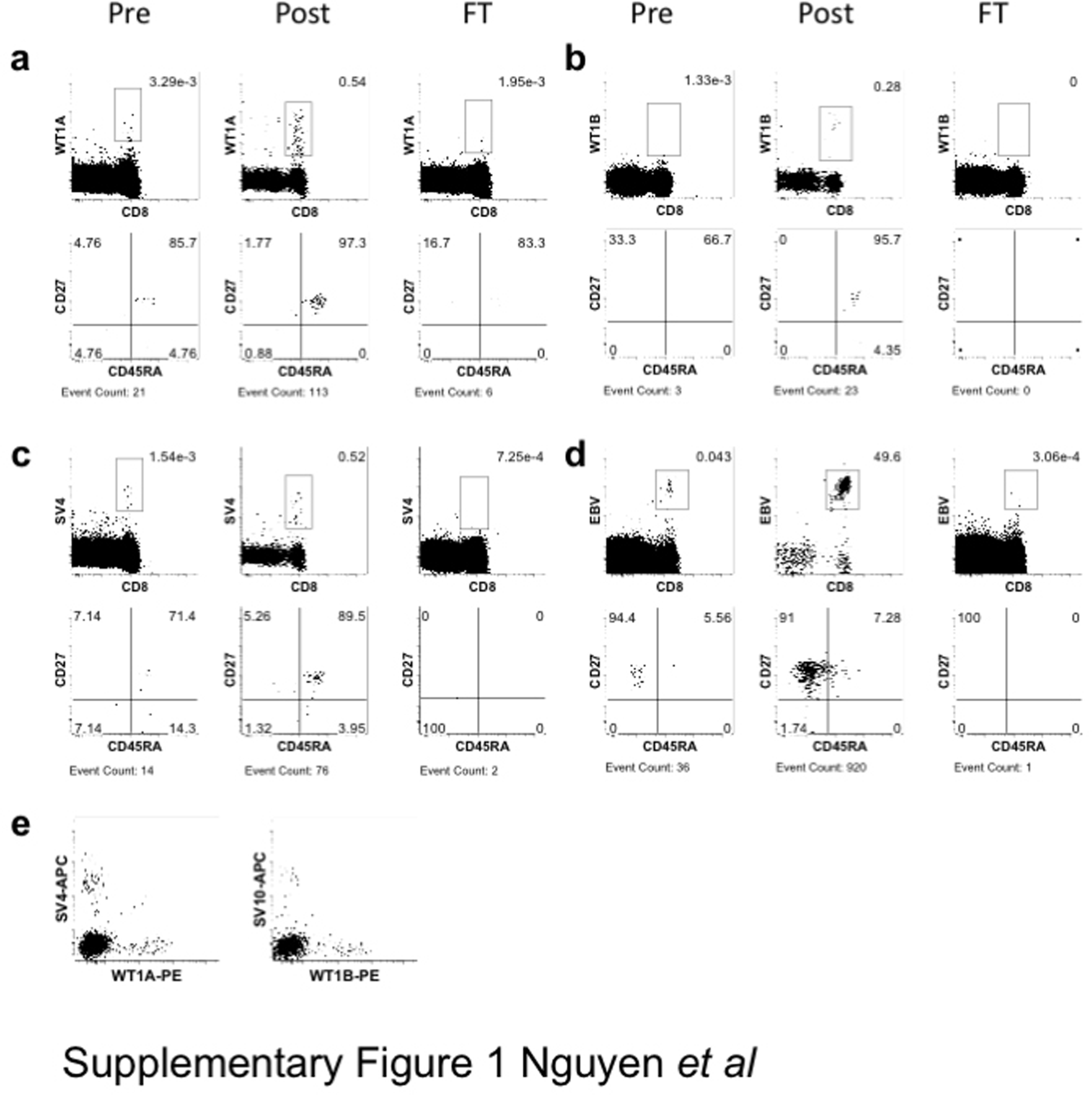

Supplement: Supplementary Figure 1 [file cti20174x2.tif]

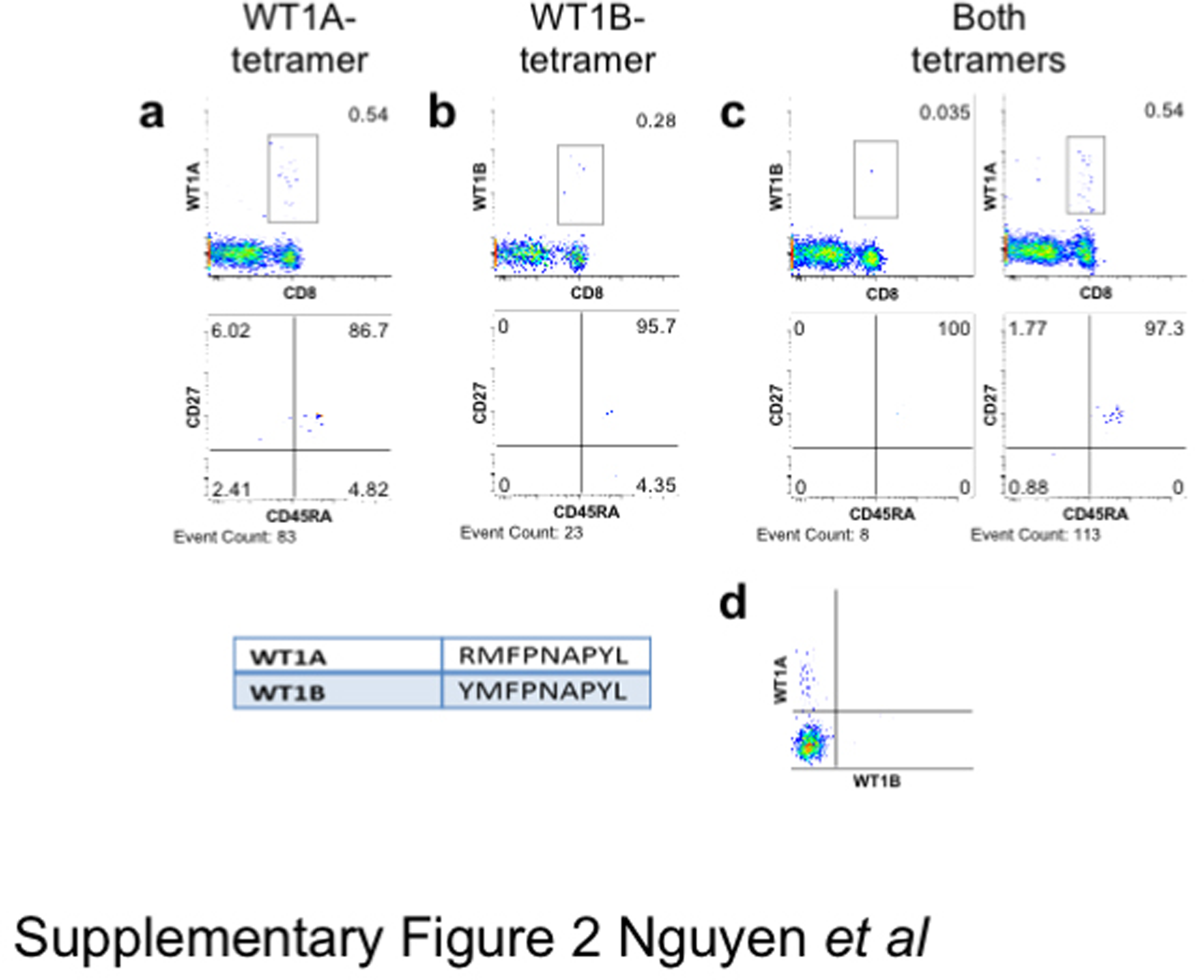

Supplement: Supplementary Figure 2 [file cti20174x3.tif]

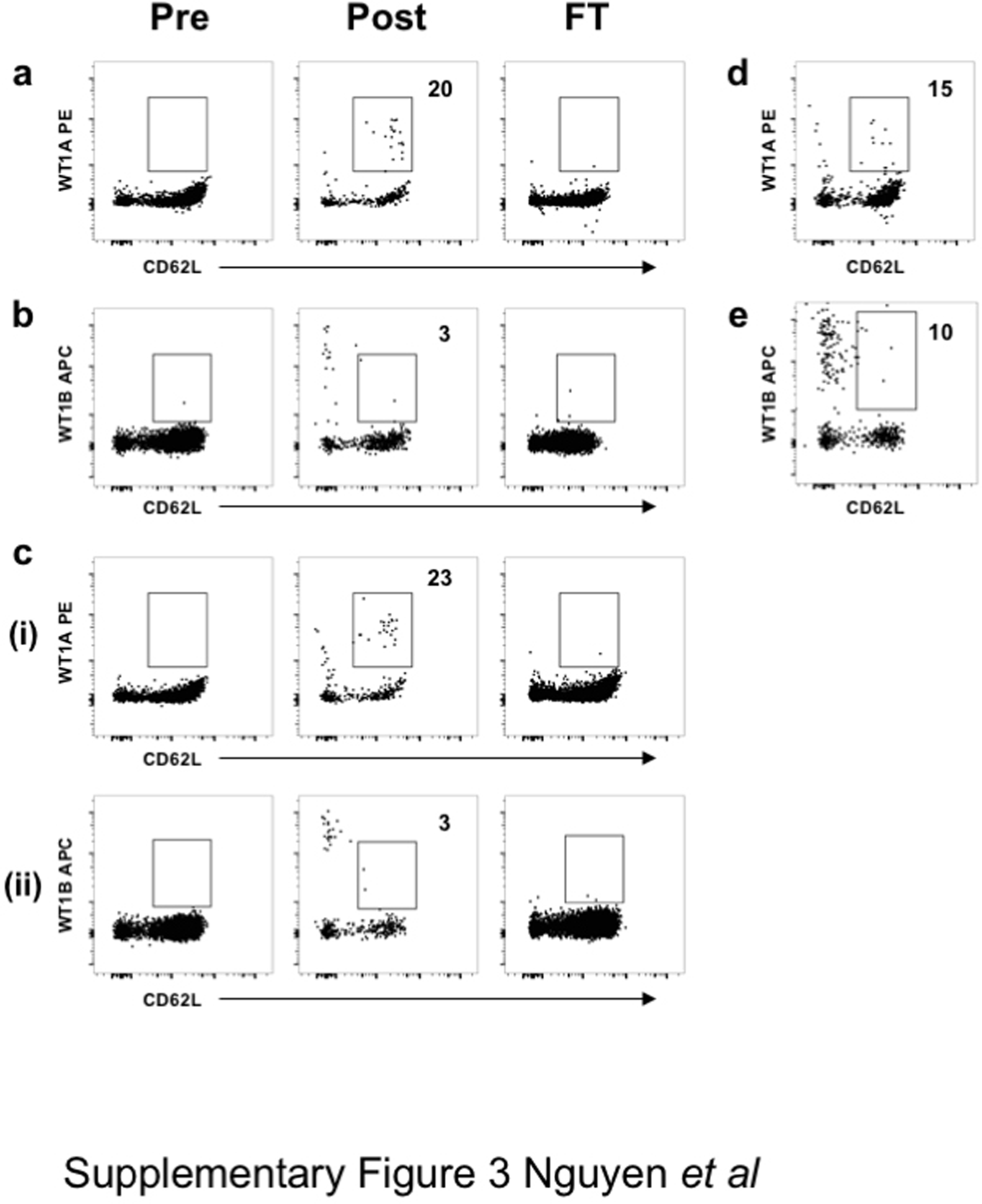

Supplement: Supplementary Figure 3 [file cti20174x4.tif]
